# Community knowledge, perceptions, and practices regarding malaria and its control in Jabi Tehnan district, Amhara Region, Northwest Ethiopia

**DOI:** 10.1186/s12936-021-03996-5

**Published:** 2021-12-09

**Authors:** Abebe Asale, Zewdu Abro, Bayu Enchalew, Alayu Teshager, Aklilu Belay, Menale Kassie, Clifford Maina Mutero

**Affiliations:** 1 International Center of Insect Physiology and Ecology, Addis Ababa, Ethiopia; 2 International Center of Insect Physiology and Ecology, Nairobi, Kenya; 3 University of Pretoria Institute for Sustainable Malaria Control, School of Health Systems and Public Health, University of Pretoria, Pretoria, South Africa

**Keywords:** Malaria prevalence, Malaria knowledge, Behavioral change, Control strategy, Ethiopia

## Abstract

**Background:**

Use of long-lasting insecticidal nets (LLINs), indoor residual spraying (IRS), community-based malaria education, prompt diagnosis and treatment are key programme components of malaria prevention and control in Ethiopia. However, the effectiveness of these interventions is often undermined by various challenges, including insecticide and drug resistance, the plasticity of malaria vectors feeding and biting behaviour, and certain household factors that lead to misuse and poor utilization of LLINs. The primary objective of this study was to document households’ perceptions towards malaria and assess the prevalence of the disease and the constraints related to the ongoing interventions in Ethiopia (LLINs, IRS, community mobilization house screening).

**Methods:**

The study was conducted in Jabi Tehnan district, Northwestern Ethiopia, from November 2019 to March 2020. A total of 3010 households from 38 villages were randomly selected for socio-economic and demographic survey. Focus group discussions (FGDs) were conducted in 11 different health clusters considering agro-ecological differences. A total of 1256 children under 10 years of age were screened for malaria parasites using microscopy to determine malaria prevalence. Furthermore, 5-year malaria trend analysis was undertaken based on data obtained from the district health office to understand the disease dynamics.

**Results:**

Malaria knowledge in the area was high as all FGD participants correctly identified mosquito bites during the night as sources of malaria transmission. Delayed health-seeking behaviour remains a key behavioural challenge in malaria control as it took patients on average 4 days before reporting the case at the nearby health facility. On average, households lost 2.53 working days per person-per malaria episode and they spent US$ 18 per person per episode. Out of the 1256 randomly selected under 10 children tested for malaria parasites, 11 (0.89%) were found to be positive. Malaria disproportionately affected the adult segment of the population more, with 50% of the total cases reported from households being from among individuals who were 15 years or older. The second most affected group was the age group between 5 and 14 years followed by children aged under 5, with 31% and 14% burden, respectively.

**Conclusion:**

Despite the achievement of universal coverage in terms of LLINs access, utilization of vector control interventions in the area remained low. Using bed nets for unintended purposes remained a major challenge. Therefore, continued community education and communication work should be prioritized in the study area to bring about the desired behavioural changes.

**Supplementary Information:**

The online version contains supplementary material available at 10.1186/s12936-021-03996-5.

## Background

Malaria is a major threat in Ethiopia with high health and economic burden, especially among poor households. It also imposes a fiscal burden on the national government due to public health expenditure related to treatment and prevention of the disease. In 2017 alone, 71 million Ethiopians were at risk, 2.7 million people were infected, and 5300 died due to malaria [1]. Thus, malaria profiling provides insights that can help design targeted strategies to reduce the impact of the disease.

Currently, Ethiopia relies on multiple interventions namely community empowerment and mobilization, vector control using LLINs and IRS, prompt diagnosis and treatment and disease surveillance to curb the burden of the disease [1]. However, insecticide resistance [2], drug resistance [3], the plastic nature of malaria vectors feeding and biting behaviour [4], and household factors hindering the proper use of LLINs [5] constitute series challenges which undermine the efficacy of various interventions. Therefore, it is imperative to explore new approaches and tools to augment the existing malaria control interventions.

One of the emerging approaches being evaluated by researchers, and malaria programmes in Africa is house screening (HS), involving the screening of open eaves, windows, and doors of houses [6–11]. HS reduces malaria among house occupants by serving as a physical barrier to mosquito entry into house. HS is not a new innovation, but it has never got enough attention as a practical intervention for malaria prevention at household level [11–14].

There are limited research reports that focus on malaria profiling in Jabi Tehnan district and their aims, delimitations and contexts also varied. For instance, Animut et al. conducted a study focused on hospital-based data to determine the causative agent of acute febrile illness in the area and reported malaria as a primary source of morbidity in the area [15]. Ayalew et al. conducted a malaria cross-sectional survey in Jiga area, which was limited to three *kebeles* and reported a 2.8% prevalence rate [16]. Further two studies were conducted by Animut et al. [17, 18]. These studies do not comprehensively reflect the malaria profile in the area as the former mainly focuses on peoples’ perception of basic malaria information. The latter mainly describes the dry season vector information in the area. This paper aims at documenting peoples’ perception towards malaria, the prevalence of the disease, and the constraints related to the ongoing interventions such as access and utilization in Northwest Ethiopia.

## Methods

### Description of the study area

The study was conducted in Jabi Tehnan district in Amhara Regional State, Northwestern Ethiopia from November 2019 to March 2020. The district population was 211,516 in 2017, with an average annual growth rate of 2.8% [19]. The district is divided into 38 *kebeles*, the smallest administrative units of the country, and has three town administrative towns. More than 90% of the people in the district live in rural areas practicing mixed farming.

**Fig. 1:**
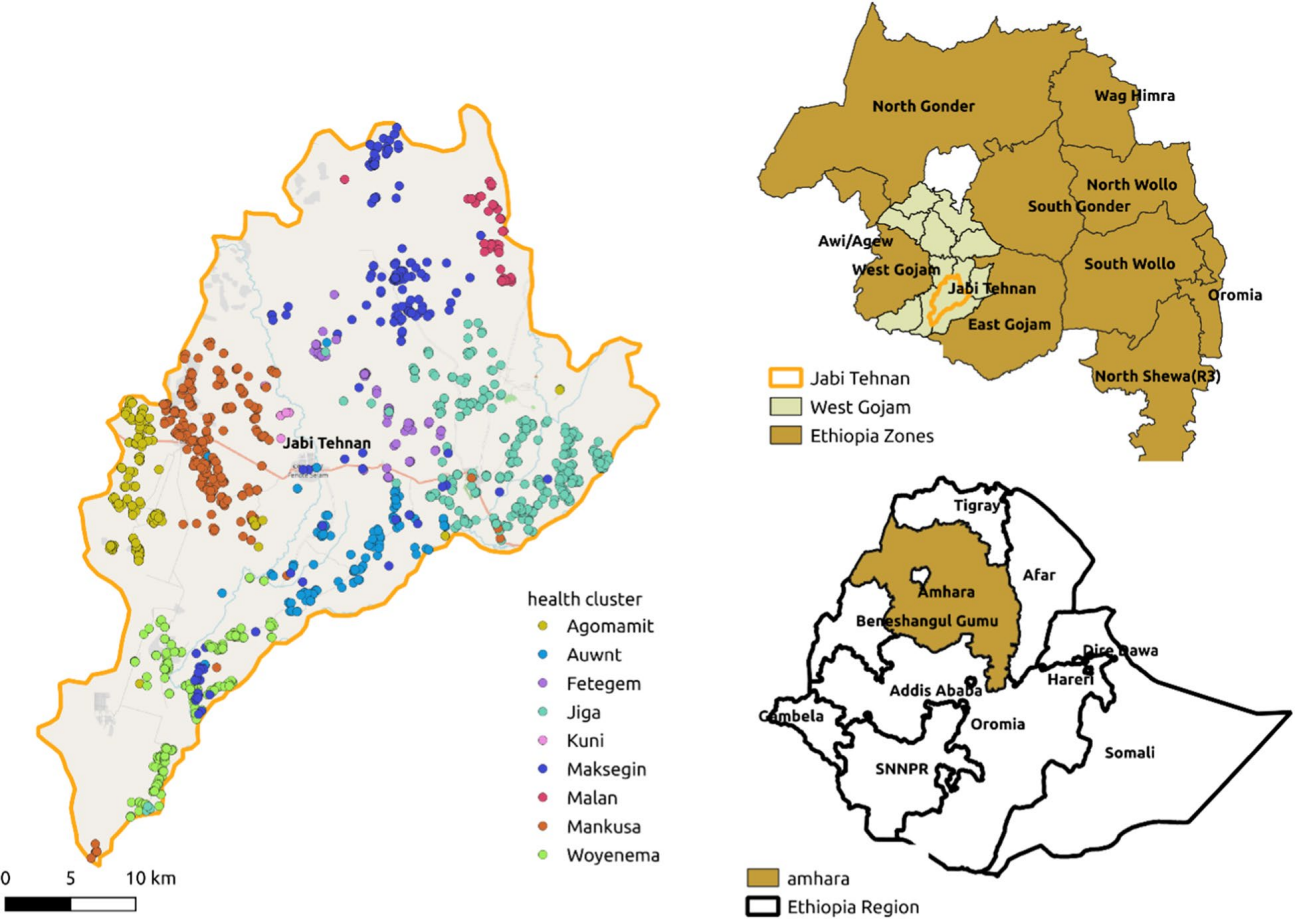
Map of the study area (Courtesy: Lea Leiman). Dots imply households visited

The district covers 1170 km^2^ at an altitude of between 1500 and 2300 m above sea level. The topography is classified as 65% flat, 15% mountainous, 15% undulating, and 5% valley [20]. The average annual rainfall is 1250 mm and has a bi-modal distribution, with the first rainy season lasting between March to April and the second lasting for 4 months from mid-June to mid-September. Minimum and maximum temperatures are 14 °C and 32 °C, respectively [20]. The weather, topography, and agroecology of the district are favorable factors for the presence of mosquito populations (Fig. 1). Mixed socio-economic activities (agriculture and animal husbandry) are practiced in the area. The area is known for keeping live stock in either close proximity or with in the human residence as it is mostly practiced in other parts of the country [21, 22].

Malaria is one of the major causes of morbidity and mortality in the district, alongside HIV/AIDS, Tuberculosis, cardiovascular, lower respiratory, and diarrheal disease [23]. Malaria prevalence in the area was 2.8% in 2013 [16]. This prevalence is higher than the average for Amhara Regional State (1.1%), where this district is situated, and the country’s average prevalence (1.2%) [24]. Despite a decline in malaria burden in recent years, the disease remains a top health threat, with annual cases surpassing 20,000 in 2017 in the district (unpublished data from District Bureau of Health). High hospital-based parasite positivity rates up to 65.4% (both clinical and microscopy combined) was reported from health facilities.

*Anopheles arabiensis* is the dominant malaria vector, while *Anopheles pharoensis* has a secondary role in transmission [18]. Both vector species are known for their zoophagic [25] and anthropophagic behaviour [21, 26]. The former species is commonly known for its opportunistic feeding and resting behaviour [25], whereas the latter is widely considered as zoophagic and exophilic species [21]. In Ethiopia, *An. arabiensis* is known for its early and late night biting activity though it continues to be active throughout the night [27–29]. According to a preliminary assessment, cattle, sheep, donkey and poultry are commonly raised domestic animals in the area.

The Ethiopian government is committed to eliminating malaria in low and intermediate transmission areas such as the Jabi Tehnan district [30]. Documenting the progress by triangulating various data sources will help understand the malaria elimination effort status and the prospect of attaining this government plan [16].

### Data collection methods

#### Household surveys

The study area had 38 *kebeles*, which were for purposes of this research further sub-divided into 115 sub-*kebeles*. For each *kebele*, up-to-date census of the chairperson and members of one-to-five groups were prepared and used to provide the required sampling frame for different surveys and FGD. The one-to-five groups are local level farmers’ organizations. Everyone is organized into a group of five people and a chairperson. These groups identify the development priorities of their respective community. They plan all the activities that need to be done in groups and individually. Every 3 days, the chairs of each group meet with the development committee of their respective kebele. Each group evaluate their performance every 15 days. The chairpersons of the one-to-five groups are the nodes of communication.

Between 3 and 56 households per sub-*kebele* were randomly selected for the surveys. The number varied from sub-kebele to sub-kebele depending on the number of one-to-five groups and the population of sub-villages. A total of 3010 households were interviewed from the selected kebeles in June and July 2019. This sample size, selected using *sampsi* Stata command [31], is required to detect the impact of the intervention. The detailed sampling strategy and the description of the project are documented in [32]. The data were collected by trained enumerators using a structured questionnaire, which included socio-economic characteristics of the households, self-reported malaria incidence, bed net ownership and utilization, the health-seeking behaviour of the households, and costs of malaria treatment.

#### Focus group discussions

The objective of the focus group discussions (FGDs) was to understand residents’ perceptions on malaria incidence in their locality. The district is divided in to 11 health clusters. Each health cluster has one health centre and 3 to 5 health posts. Therefore, we conducted one FGD per cluster. Thus, a total of 11 FGDs were undertaken in 11 health clusters of the district, which were purposively selected to reflect agroecological differences and information coverage across the district. The participants of the FDGs were 7–10 people per FGDs. The FDGs were undertaken by three members of the research team. The first member was responsible for leading discussion while the second researcher was tasked with taking notes. The third member recorded the discussion. All discussions and interviews were conducted based on a prepared semi-structured interview focusing on malaria knowledge, malaria control interventions and the role of indigenous knowledge in malaria control. The FGDs were held in Amharic, which is the working language of the Federal Government of Ethiopia and the mother tongue in the study area. In addition to the FGDs with the community members, local health workers were asked questions on the awareness of the communities regarding malaria illness, communities’ health-seeking behaviours, common vector control interventions practiced in the area, how they performed diagnosis and treatment of patients, and challenges in malaria control and elimination in the district.

#### Parasitological screening

This involved a cross-sectional malaria parasitological survey to estimate *Plasmodium* parasite prevalence, transmission intensity, identification of specific *foci* of local transmission and identification of the *Plasmodium* species presents in the study area. This would potentially allow programme managers to prioritize severely affected villages for malaria control interventions, amid limited logistical capacity and shortage of professional experts [33, 34].

Information from the baseline household survey data was used to select individuals for the parasitological survey. The parasitological screening was carried out from households with at least one child aged 10 years and below. In this study, one child per household was tested. In households where more than one child under 10 was encountered, one child was randomly tested. The focus on this age cohort is because they are at higher risk of malaria than other people. This age cohort is also less mobile, which may reflect the true disease prevalence [35]. GPS coordinates of houses of each child were taken using a handheld GPS unit and study villages and households of all children were mapped.

Blood samples were collected in November and December 2019. A finger prick blood sample was collected after cleaning the finger surface using sterile cotton wool soaked in 70% ethanol by trained technicians. The thick smear was served to confirm the presence or absence of *Plasmodium* parasites, whereas the thin smear was fixed with methanol and stained with Giemsa (3%, PH, 7.2, for 20 min) to identify the parasite species. Microscopic examination was conducted under 100× magnifications, and 100 fields were examined before a negative result was confirmed. All blood films were initially read on-site or at local malaria control laboratories by trained laboratory technicians. Films positive for parasites and a 10% sample of films negative for parasites were subsequently checked by an independent senior laboratory technician at Finoteselam Hospital Laboratory. The parasite density (parasite/mm^3^) was calculated by assuming an average leukocyte concentration of 8000 leukocytes/mm^3^ [36]. Thus, the recorded parasite density was obtained by multiplying the observed number of parasites by 25. Prevalence of single and multiple species of parasites was assessed and classified by gender and age groups. χ^2^ test was used to compare the burden between gender and age group. P-value of < 0.05 at 95% CI was used to test the statistical significance between the dependent and independent variables.

### Data analysis

Demographic variables such as marital status, education, occupation, housing setting were presented using descriptive analysis. However, variables were further dichotomized between households that reported malaria and not reported malaria to determine the indicator variables. Thus, difference between households that reported malaria and not reported malaria were presented using percentile. Malaria treatment-seeking behaviour and possible sources of treatment in the area were presented using percentile. Line graphs were used to indicate trends of malaria case in study villages across villages, years, and specific months of the year.

### Ethical clearance

The stakeholders, *kebele* management and health sector (including zonal, district and kebele) workers in the study area were informed about the objective of the study. Consent was obtained in advance from heads of households that took part in this study. The objective of the study and participants’ right to quit at any step from being part of the study was explained before the commencement of data collection. Data collectors that took part in blood sample collection were separately trained. Study subjects found positive for malaria infection during this survey were promptly treated according to the national malaria treatment guideline [37]. This study was cleared by the ethical committee of Amhara Regional State Public Health Institute, and submitted to the zonal, *woreda* and *kebele* administrations.

## Results

### Socio-demographic characteristics of the study subjects

The socio-demographic characteristics of the study village are presented in Table 1. Most of the households (89%) were led by males and a similar majority (88%) were married. More than half (55%) of the respondents were illiterate. The overwhelming majority (98%) of heads of the households were farmers. Each household had an average family size of 5.6. A large proportion (30%) of the interviewed households reported having no bed nets. The number of bed nets owned by the households (1.64) was quite small relative to the average size of the households. Houses were made of wooden walls and plastered with mud. House roofs were made from corrugated iron sheet, with 57% of them without eave gaps. The eaves of the remaining 43% of houses were partially or fully open. Nearly 10% of respondents also reported that their houses were screened so that mosquitoes could not enter the house in the evening. More than half (56%) of the community had separate cattle sheds or houses. However, 40% of the households reported that they sheltered animals within the houses.

**Table 1:** Socio-demographic characteristics of the study subjects

Classification	Variable (1 = yes, 0 = no)	Households reported malaria		Households reported no malaria		Mean difference	P-value
		Mean	SD	Mean	SD		
Gender	Sex of the household head is male (1/0)	0.89	0.31	0.84	0.37	0.05	0.00
Family size	Family size	5.3	1.92	4.75	1.92	0.55	0.00
Marital status	Married (1/0)	0.88	0.33	0.83	0.38	0.05	0.00
Education	Illiterate (1/0)	0.53	0.50	0.57	0.5	− 0.04	0.02
	Schooling 1–7 years (1/0)	0.41	0.48	0.36	0.48	0.05	0.00
	Schooling above > 7 years (1/0)	0.07	0.25	0.08	0.27	− 0.01	0.40
Occupation	Farming (1/0)	0.98	0.14	0.99	0.12	− 0.01	0.21
Bed nets	Have bed nets (1/0)	0.70	0.46	0.70	0.46	0.00	0.94
	Number of bed nets owned	1.64	0.76	1.64	0.7586	0.09	0.86
	Number of rooms with bed nets	1.83	1.79	1.63	1.42	0.20	0.00
Housing characteristics	Eave open (1/0)	0.18	0.38	0.16	0.37	0.01	0.34
	Eave fully closed (1/0)	0.53	0.50	0.6	0.49	− 0.07	0.00
	Eave partially closed (1/0)	0.29	0.45	0.23	0.42	0.06	0.00
	Main house screened (1/0)	0.11	0.31	0.09	0.29	0.02	0.10
	Animals sheltered within house (1/0)	0.42	0.49	0.4	0.49	0.02	0.28
	Animals sheltered outside house (1/0)	0.53	0.49	0.53	0.5	0.00	0.89
	No animals (1/0)	0.05	0.21	0.06	0.24	− 0.02	0.04
	Number of observations	3010					

### Malaria testing and treatment seeking behaviour

Malaria treatment-seeking behaviour and economic impact due to the disease are presented in Table 2. Thus, 14% of the respondents have reported that they were ill because of malaria in 2018/19 and it took them on average 4 days before they report the case to the nearby health facility. The number of working days lost due to malaria illness in the area was reported to be on average 2.53 per person-per episode and the amount of money spent per person-episode was 18.18 USD. These costs included both direct (medical expenses) and indirect (transport, meal). A labourer in Ethiopia earns a wage of approximately 170 to 350 ETB, i.e., translated in USD as 4.5 to 8.75. Thus, the working days lost per episode per person was estimated to be between 11.4 and 22.14 USD.

**Table 2:** Reported malaria treatment-seeking behaviour and economic impact

Variable	Mean	SD	Min.	Max.
Members were ill because of malaria in 2018/19 (1/0)	0.14	0.34	0	1
Number of days lost due to sickness/person	2.53	5.36	0	90
Days before seeking treatment?	3.96	7.30	0	90
Total out of pocket expenditure (USD)	18.18	26.73	0	213.79

Different treatment sources used by the community members in the study area are presented in Table 3. Village clinic or health post and hospitals were reported to be the main source of treatment according to 91% of the respondents. There were a small portion of the respondents (1.6%) who reported to use traditional healers exclusively and another small portion of respondents (7.66%) who did not seek any treatment.

**Table 3:** Reported treatment sources in the study area

Places	Freq.	Percent
Village clinic	1507	73.01
Hospital	243	11.77
Traditional healer	33	1.6
Village clinic and hospital	92	4.46
Village clinic and traditional healer	16	0.78
Hospital and traditional healer	11	0.53
All	4	0.19
Did not seek treatment	158	7.66

### Results from focus group discussion

#### Participant’s’ malaria knowledge

Under this section participants were asked to discuss and reflect the general information about the presence and absence of malaria disease in their villages, malaria disease trend in the last 5 years, the estimated number of people who became sick within their family and/or within their community, the most affected group of people, common place of work of community members in the area, time of work and malaria calendar. Accordingly, all participants of the FGD were in agreement that there were active malaria cases in their villages. However, participants underlined a significant decline in malaria cases in recent years across the villages (Table 4).

**Table 4:** Reported malaria disease knowledge from FGD groups, Jabi Tehnan district, NW Ethiopia

Health cluster	Estimated malaria cases/year	Affected group	Mosquito biting time	Malaria calendar
Fetegem	2–3	Children, night guard workers, farmers	Night	April–September
Maksegn	4–15	Children, pregnant women, farmers	Night	April–September
Kuni	10–15	Children, pregnant women, farmers,	Night	April–September
Agomamit	4–100	Children, pregnant women, farmers	Night	April–September
Mankusa	15–50	Children, pregnant women, farmers	21:00–23:00	Year round
Woyenema	Difficult to estimate	Children, people work in irrigated fields	21:00–00:00	Year round
Awunt	Difficult to estimate	Children, people work in irrigated fields	Night	Year round
Jiga	5–20	Children, people work in irrigated fields	Night	April–September
Yeraber	Difficult to estimate	Children, pregnant women	Night	April-September
Malan	Difficult to estimate	Children, pregnant women	Night	April–September

FGD participants from Fetegem, Maksegn, Kuni, Agomamit, Mankusa and Jiga estimated figures of malaria cases in their community. Accordingly, the least estimate (2 to 3 cases/year) was reported from Fetegem, and the highest estimate 100/year was reported from Agomamit. Despite the presence of malaria in their villages, FGD participants from Woyenema, Awunt, Yeraber and Malan indicated that they could not put forward the exact or estimated number. Malaria was transmitted through mosquito bites that occur during the night according to all participants. Malaria was reported to seasonal, occurring between April to May and Sept to November. Participants from Woyenema and Awunt however, reported that malaria occurred all year-round in their village. One respondent from *Fetegem* health cluster raised a very important point about how people who work in the security sector (community policing members, private security guards) were disproportionately affected by the disease. According to the respondent.

*“People who work in night security shifts usually get tired at some point and fall asleep in unguarded places (security huts) where they get mosquito bites and acquire the disease.”*

A respondent from *Maksegn* health cluster also added other risk factors observed in their area. These included the habit by some community members who stay outdoors until 19:00 h, keeping livestock outside the main home and guarding them in the night which leads to infection by malaria vectors.

#### Participant’s access to and utilization of malaria control intervention

The discussion under this heading focused on curative and preventive interventions in respective localities within the study area. The FGD participants agreed that LLINs were the only preventive intervention available in all the health clusters and IRS was available only in four villages (*Fetegem*, *Jiga*, *Woyenema* and *Yeraber*) of the district. According to the participants filling and draining of standing water was the most widely practiced supplementary intervention used to remove mosquito breeding habitats.

Despite these community and government-led efforts, there were still challenges to eliminate malaria in the study area. For instance, some community members dug water reservoirs for different purposes including preparation of mud for wall plastering and left the pits undrained. Some individuals also constructed houses for rental purposes and kept them closed for a long period of time. These uncontrolled practices of some community members often created suitable breeding and resting places for mosquitoes respectively. The other key challenge observed by FGD participants was repurposing of bed nets. Participants said that it was a common practice to see people using bed nets as bags to transport goods, repurposing them as ropes, or using the nets to cover grain stores or barley heaps (Additional file 1: Plate S1; Additional file 2: Plate S2).

Despite the overwhelming majority of FGD members noting that people can get services from health facilities which are in their own villages, on average found within a 5 km distance, one participant from *Maksegn* health cluster and another from Woyenema stated that people travelled between seven to 20 km to seek treatment.

#### Participant’s indigenous practices in malaria prevention and control

Under this section, the study team probed the FGD participants on different cultural (e.g., traditional medicines) and religious practices (e.g., spiritual rituals). Participants from *Fetegem* health cluster stressed the predominance of modern medicine for treatment in their area but they also reported the practice of making extracts from leaf and succulent parts of *Ocimun lamiifolium* (local name: “*damakese*”) in the past. Participants from *Agomamit* health cluster reported the practice of eating mashed garlic. In the same way participants from *Maksegn* health cluster revealed there was a practice of using leaf extracts from *Phytolacca dodecandrian* (“*Indod*”), *Clausena anisate* (“*limmich*”). In *Awunt health* cluster, FGD participants reported that they used a mix of honey, garlic, *and Croton macrostachyus *(local name: “*Bisanna*”) extracts*.*

### Results of the parasitological screening

**Fig. 2:**
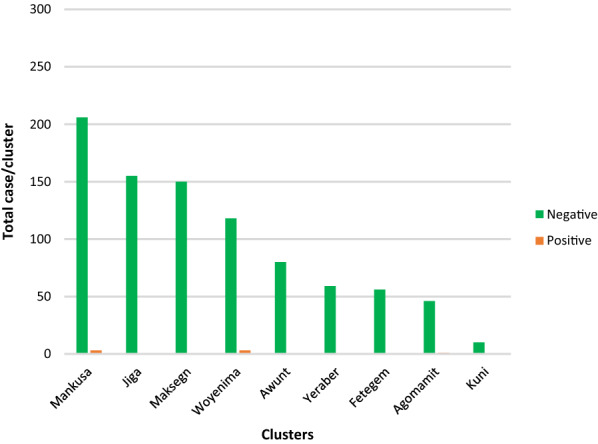
Malaria prevalence in different health centres in the study area (2019)

Out of the 1256 randomly selected subjects who gave their consent for the malaria parasite test, 11 (0.89%) were found to be positive (Fig. 2). Out of the 11 positive children, 6 were found in the Mankusa cluster (Goref and Abasem kebeles), 4 were found in the Woyenema cluster (Ergib Kebele), and 1 was found in Agomamit cluster (Guay kebele). Out of the 11 malaria cases identified 9 (82%) of the samples were identified as being due to *Plasmodium vivax*, while the remaining 2 were *Plasmodium falciparum*.

**Fig. 3:**
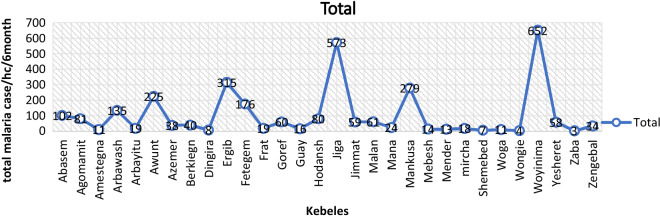
Malaria case reports from health facilities by village in the study area (July–December 2019)

According to hospital and clinic-based reports in JabiThenan district, there were a total of 3315 malaria cases documented in the months between July and December 2019. Woyenema (652 cases), Jiga (573 cases), Ergib (315) and Awunt (225 cases) were the top four malarious villages documented in the district. *Plasmodium falciparum* was more prevalent with a total number of 1885 (57%) as compared to *P. vivax* which accounted for 1244 (37.5%) (Fig. 3).

**Fig. 4:**
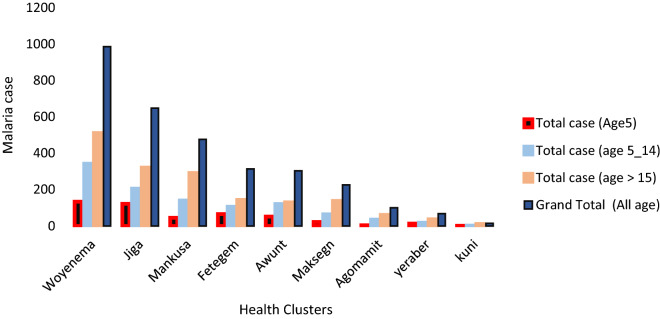
Malaria burden in different age groups reported from health facilities in the study area

Malaria disproportionately affected the adult segment of the population as 50% of the total cases were reported from people whose age was 15 years and above. The age group between 5 and 14 years was the second most affected group followed by children under 10 with 31% and 14% of the cases for each group, respectively (Fig. 4).

### Trend analysis

**Fig. 5:**
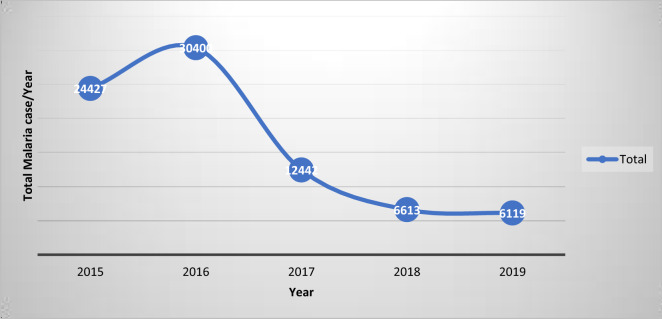
Trend analysis of malaria cases reported in Jabi Tehnan district Northwestern Ethiopia (2015–2019)

Overall malaria cases in Jabi Tenan district were observed to substantially and sequentially decline during the 5 year period from 2015 to 2019. Thus, the highest number of malaria cases (30,400) was documented in 2016 and the lowest number (6119) in 2019. This shows that there was 80% reduction of cases during the period. However, a comparison of the last 2 years data (2018 and 2019) showed a stagnation in case reduction (Fig. 5).

## Discussion

Malaria control programme managers mainly depend on case reports from health facilities to make major intervention decisions [30]. The fact that decisions are made mainly based on case reports puts the entire programme on passive posture, i.e. if other outbreak alert and response methods such as entomological information, weather forecast, regular cross sectional malaria indicator surveys are not in place, the programme office will always left with to play catch-up to contain the disease outbreak [38]. While case reports from health facilities always remain a critical component in deriving both policy and strategic decisions, they may not sufficiently provide the necessary information regarding malaria transmission intensity, hot spots, and endemicity problem. In this study, key malaria disease control indicators were assessed in Jabi Tehnan district using household level KAP and cross-sectional malaria parasitological surveys. In addition, recent trend of malaria cases and its implication for malaria elimination was evaluated.

The socio-demographic features of community members in the study area were similar to those reported from other parts of Ethiopia including a pre-dominance of male-led households, high level of illiteracy and engagement in agriculture as the main community occupation [39]. The average family size of 5.6 reported here is slightly higher than the country average of 4.6 [19]. It might be economically difficult for large households to buy enough insecticide-treated bed nets and this is documented in studies conducted in Gambella and Jimma, Southwestern Ethiopia [40, 41]. LLINs are freely available by the government in Ethiopia, however, a household cannot receive more than four LLINs regardless of any extended family size. This means households with extended family size are forced to buy the extra bed nets needed. Most members of the community have separate cattle sheds or houses, and this is an important behavioural difference documented compared to the reluctance of families in most parts of Ethiopia to separate cattle from human residences. Keeping livestock away from human residences is acknowledged as a practical strategy for diverting certain malaria vector species from human hosts to other vertebrate hosts, thereby, decreasing the contact between humans and infectious mosquitoes [42, 43]. Houses in this study were made of walls with wooden frames, plastered mud walls and roofs made of corrugated iron sheets. The absence of traditional huts with grass thatched roofs could be partly explained by improved economic status of the community [44]. The coverage of households in the area with LLINs was found to be moderate, with every household having at least 0.5 LLINs and with total coverage being 70%. This was below the country and regional average of 72% and 76% respectively [24].

Both long-lasting insecticidal nets (LLINs) and indoor residual spraying (Propoxur, 1–2 g m^2^) were being used in the district with IRS given priority to 4 villages (Ergib, Awunt, Hodansh and Jiga Yelimdar) which had higher ongoing transmission. LLINs was the only intervention available throughout all the health clusters of the district. Universal coverage (100% access) was ensured in the district as confirmed through FGD, personal observation and information obtained from the district health department. Supplementary interventions such as filling and draining of standing water were reported by FGD participants even though they were not regularly implemented. Major emphasis was given to case treatment and bed net distribution from malaria control programme office of the district.

Universal coverage or access to at least one vector control intervention/per household is the direction adopted by national malaria control programme since 2006 in Ethiopia [45]. Low utilization of the already available interventions and using nets for unintended purposes however remains major challenges in the area as close to half (50%) of the households who had access to bed nets did not use it in the previous nights in the area. Lack of persistency in using bed nets is a cross-cutting problem throughout the country as it is assessed in country wide malaria indicator surveys [24]. Continued community education and communication effort should be done in order to bring the desired behavioural changes.

As it is confirmed in this study repurposing of bed nets was reported as commonly available. Thus, there is serious gap in behavioural change to be addressed from all stakeholders involved in malaria control programmes. Studies documenting misuse of bed nets in Ethiopia are not many, but a study conducted in Adame-Tullu district of East Shewa zone, central Ethiopia showed that misuse and repurposing of bed nets for other purposes such as wrapping maize kernels, wrapping teff and transporting it from field to home using animal carts are the common acts of misuse [5]. The observed repurposing of LLINs can be improved through continued teaching of communities about the adverse effects of accidental contamination of cereals covered with insecticidal nets and facilitating access of target communities to cheaper and suitable materials for making ropes, transportation, or coverage of grain stores.

In this study, different traditional practices were assessed, including vector control and diseases treatment approaches used by the communities in the study area. Despite the reliance on modern medicine predominantly as the participants described, some community members believed that extracts from leaf and succulent parts of different plants such as *Ocimun lamiifolium* (loc. “*damakese*”), *Phytolacca dodecandra* (“*Indod*”), *Clausena anisate* (“*limmich*”), *Croton macrostachyus* (“*Bisanna*”) and mashed garlic could be used for treating malaria. Provision of traditional medicine for malaria remedy is a long-established trend in Ethiopia [46, 47]. While the essence of indigenous knowledge is undoubtedly important in the quest of new antibiotic options, the anti-malarial potential for the plants has not scientifically established yet.

The delayed treatment-seeking behaviour observed in this survey is also a common problem documented throughout the country with reports from Central [48], South East [49], Western [50] and Southwestern Ethiopia [51]. While the cessation of death due to malaria in Jabi Tehnan district is an achievement, the disease continues to cost considerable working days (2.53 per person-per episode) due to morbidity and money spent on treatment (18.18 USD/person/episode) and loss of income especially for people working on daily wager. The economic impact of malaria is analysed from different perspectives including the death of workers, school absenteeism, loss of family members’ time due to caring, loss of savings, loss of household and farm assets [52]. The reported working days lost due to malaria is relatively low as compared to recent studies conducted on malaria economic impact in Ethiopia which estimates loss of 6.3 working days, but money spent per episode per person was relatively high as compared to the 17.8 USD expense/episode/person [53] in Ethiopia and Kenya with loss of 5 USD [54].

Health posts and government hospitals were reported to be the main source of treatment in the area. Malaria treatment is freely accessible in Ethiopia through government health institutions (Health Posts, Health centres, and hospitals) [1]. Despite free access to malaria treatment at every village level in the district and in other parts of the country, some people either did not seek treatment at all (7.66%) or used other unreliable treatment sources such as traditional healers (1.6%). This showed that there are still significant gaps in the awareness level of the community in treatment-seeking behaviour. However, the fact that some community members use traditional treatment options consciously for different reasons cannot be ruled out. Avoiding travelling longer distance, seek of cheaper cost, having bad experience from medical centres in the past, influence of local traditional healers are some of the justifications reported elsewhere [55].

Malaria disease prevalence in Jabi Tehnan district was low (0.89%). In this survey, *Plasmodium vivax* was the main parasite documented in the area followed by *Plasmodium falciparum*. Besides, all positive samples were documented in areas well below 2000 m above sea level, between 1300 and 18,000 m. As Jabi Tehnan is one of the 239 districts selected for all human to human malaria elimination in the country [56], the current low prevalence rate gives hope for the envisaged elimination programme. However, the recent resurgence of *P. vivax* in some of the villages (Ergib, Goref, Abasem, Jiga Yelimdar, and Guay) may jeopardize the plan.

Malaria case reports from health facilities in the area were collected concomitantly to make comparisons and cross-validations with prevalence data. Accordingly, those villages where positive cases were found in the cross-sectional survey had also the highest share of cases as reflected in clinic-based data in the district. Jiga and Awunt had reported considerably higher cases with the former being the second and the latter being fourth most affected in the district. However, no positive case was found in the cross-sectional parasite survey. Moreover, malaria disproportionately affected the adult segment of the population as 50% of the total case was reported from people whose age was 15 and above followed by the age between 5 and 14. In Ethiopia, malaria transmission is unstable and highly seasonal with few exceptions of areas bordering Sudan and South Sudan. This has resulted in low host immunity and risk of the adult population being more affected unlike the trends observed in other parts of Africa [1, 57].

In this study, malaria parasite testing was done using microscopy only. This might have contributed to the observed low level of parasite prevalence. Conventional diagnostics such as microscopy and RDT have led to missing nearly half of the asymptomatic *Plasmodium* reservoir, which were detected by more sensitive molecular diagnostic tools such as nPCR and qPCR [58, 59]. Thus, future parasite screening surveys should consider utilization of combination of both microscopy and molecular screening techniques.

Overall malaria cases have been substantially declined in the last 5 years in Jabi Tehnan district. Thus, the highest malaria case was documented in 2016 and the least case was documented in 2019. There was 80% reduction of cases in the last 5 years, however, comparison of the last 2 years data (2018 and 2019) showed that case reduction had almost flattened. Outdoor transmission in Jabi could be driving substantial portion of transmission as the area is known for harbouring large mechanized farms, such as Bir Sheleko mechanized farm and Bir Sheleko military training campus. These sites become a point of attraction for migrant workers which mostly originate from less immune highland areas [60, 61]. The temporary workers in these areas are usually stay in poorly constructed temporary shelters, which are called “satera”, tent like structures which are made of wooden framework and partially covered with plastic cover. These structures are porous and easily allow the influx of vector mosquitoes. Moreover, the structures are constructed to accommodate multiple workers and, therefore, it is difficult to put on bed nets. This was confirmed from FGD conducted by the community members and the members agreed that temporary workers, guards, people who work until late evening are the main victims of mosquito bite. The government of Ethiopia mainly focuses on aggressive deployment of indoor based vector intervention tools (IRS and LLINs), however, the loophole created by the above conditions continued to reverse hard earned gains through indoor vector control interventions and need immediate action from policy makers. This is also a common problem across Africa as a certain portion of malaria vectors defy the existing vector control efforts [45]. It becomes clear that even with universal coverage of mainstay vector control interventions, there will be still sustained transmission due to outdoor transmission [62]. Mosquito net utilization is affected by a number of factors. These include the absence of sufficient mosquito nets, the size of the net (single, double, family) [63], education level of the user, housing setting [64], sleeping and mobility patterns of the specific community [65, 66]. Thus, mosquito net distributions should be followed by appropriate operational researches in order to determine mosquito net utilization and the constraints against the achievement of the desired behaviour.

## Conclusion

In conclusion, this study assessed the status of key malaria control interventions namely access and utilization rate of LLINs, and treatment-seeking behaviour. In both regards, there was a serious gap that must be addressed through social mobilization and education. Both cross-sectional and hospital-based positivity rate studies showed that malaria prevalence in the area was low. However, the fact that the rate of reduction did not change for the last 2 consecutive years showed that there should be a concerted effort to further drive case reports to zero. The situation, therefore, calls for the implementation of supplementary interventions such as house screening as recommended in several recent studies in both Ethiopia and other countries [67–71].

Despite the achievement of universal coverage in terms of LLINs access, utilization of vector control interventions in the area remained low. Moreover, using bed nets for unintended purposes was a major challenge. Consequently, continued community education and communication would be necessary in the study area in order to bring about the desired changes in community behaviour and practices. Community education could be more effective if delivered in more targeted approaches such as malaria education for women groups, intensified education in hotspot neighborhoods and cascading malaria education through primary schools [72, 73]. Moreover, behavioural changes may take long time (years) to achieve. Hence, positive communication approaches should be implemented to slowdown community fatigue development. These include integrating passive and active mass education [74, 75], using local approaches such as coffee ceremony [53] and artistic interventions such as songs, drams and poets [76].

In this study, it was confirmed that botanical extracts from leaf and succulent parts of different plants such as *Ocimun lamiifolium* (loc. “*damakese*”), *Phytolacca dodecandra* (“*Indod*”), *Clausena anisate* (“*limmich*”), *Croton macrostachyus* (“*Bisanna*”) were being used for treatment of malaria. In addition, there were other plants being used for malaria treatment, but their names were concealed from disclosure by the healers. Therefore, the potential of these plants should be further investigated in order to identify and evaluate their active ingredients and their effectiveness for preventing or clearing malaria.

## Supplementary Information

**Additional file 1: Plate S1.** Intact bed net is being used for unintended purpose such as transporting bag for crops from field to home (Photo courtesy: Abebe Asale).

**Additional file 2: Plate S2.** Intact bed net is being used for unintended purpose such as bag for animal feed or hay near home (Photo courtesy: Abebe Asale).

## Data Availability

The datasets used and/or analysed during the current study are available from the corresponding author on reasonable request. All data generated or analysed during this study are included in this article and its Additional files.

## Abbreviations

LLINs: Long-lasting insecticidal nets
ITN: Insecticide treated net
IRS: Indoor residual spray
NMCP: National Malaria Control Programme
WHO: World Health Organization
