# Supplementary figures and images for: Community knowledge, perceptions, and practices regarding malaria and its control in Jabi Tehnan district, Amhara Region, Northwest Ethiopia
Source: Malar J. 2021 Dec 9;20:459. doi: 10.1186/s12936-021-03996-5 (PMC8656029; doi:10.1186/s12936-021-03996-5)

Plate S1


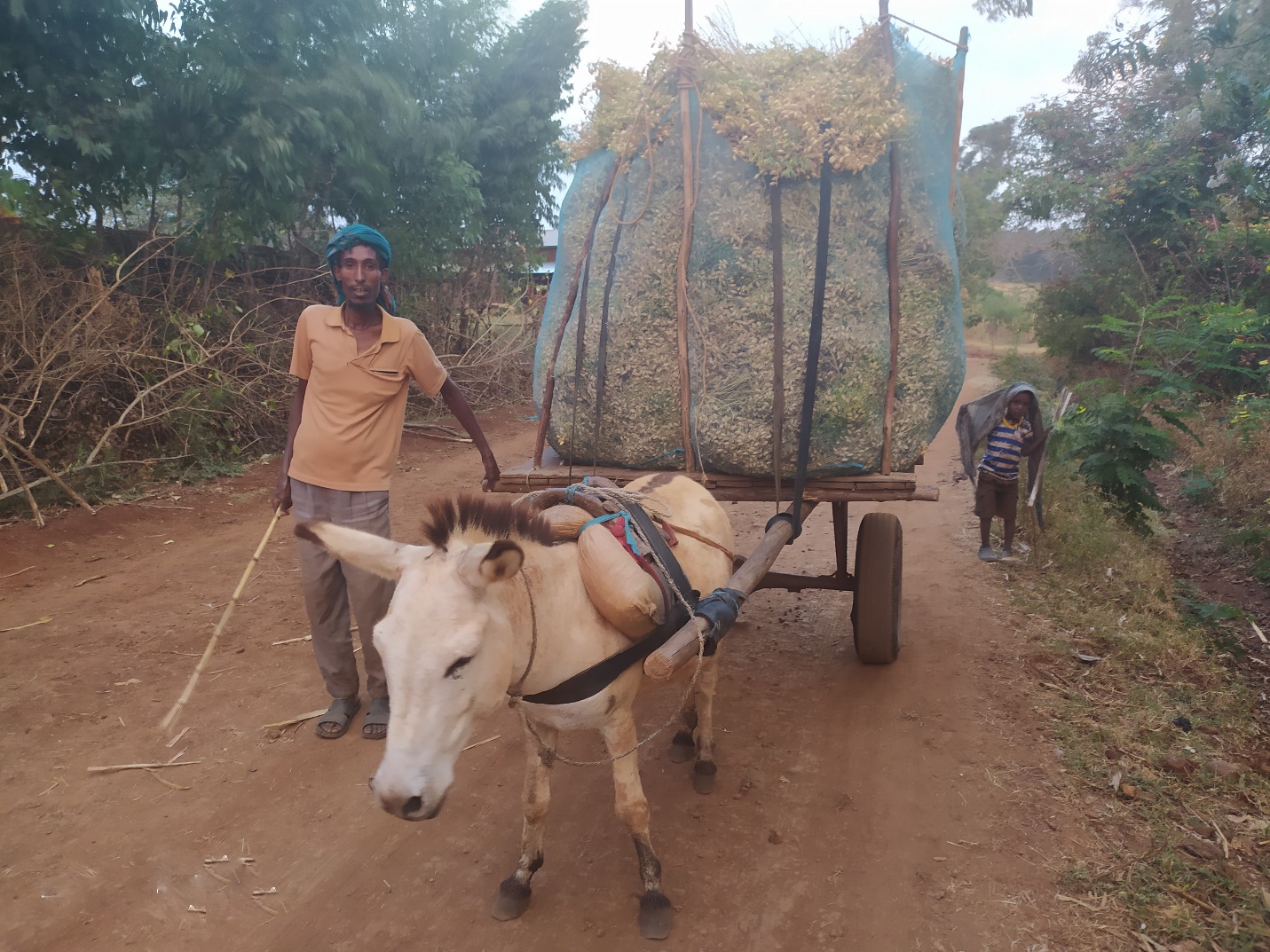

Supplement: Supplementary file 1 — Additional file 1: Plate S1. Intact bed net is being used for unintended purpose such as transporting bag for crops from field to home (Photo courtesy: Abebe Asale). [file 12936_2021_3996_MOESM1_ESM.docx]

Plate S2


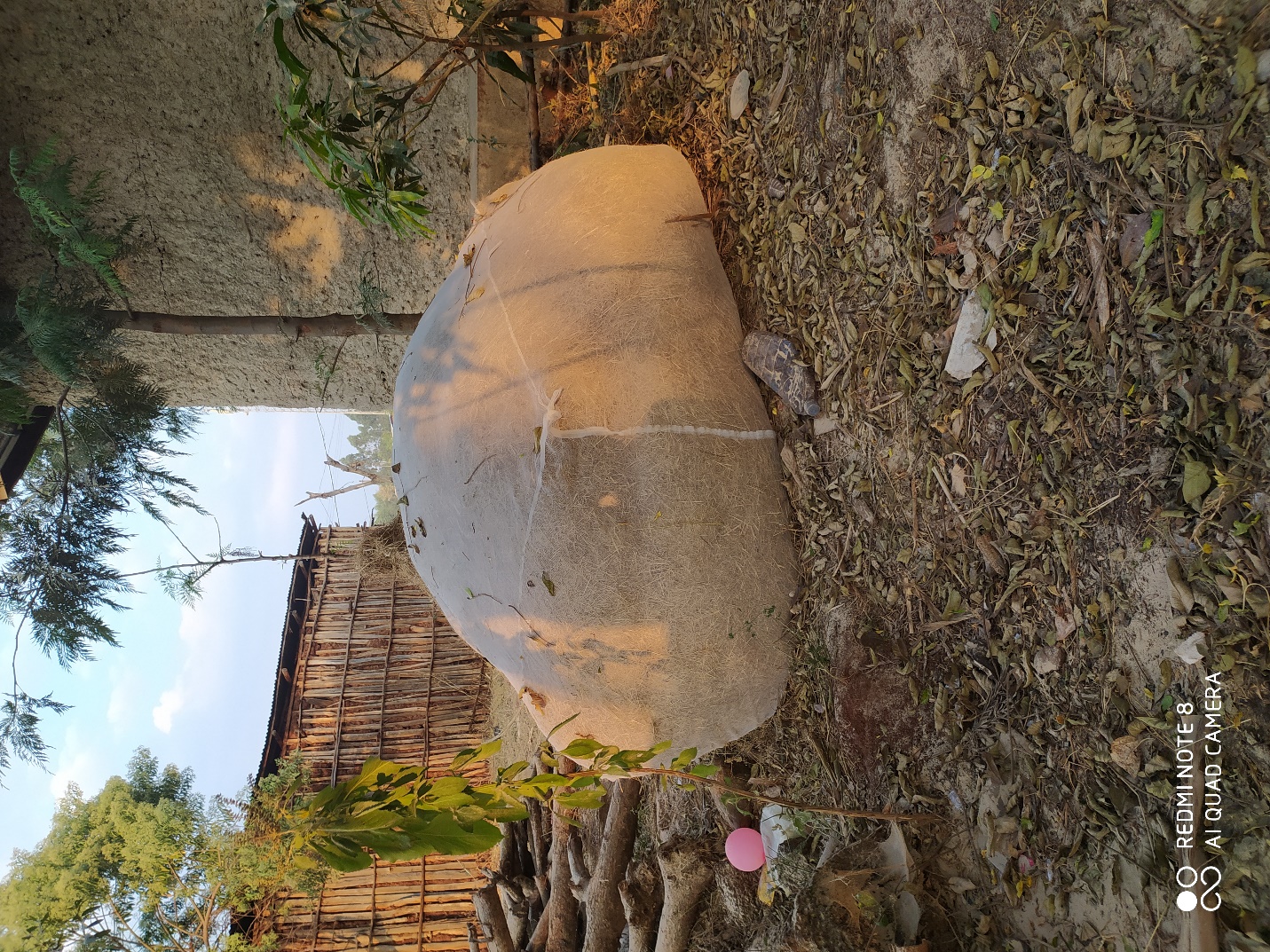

Supplement: Supplementary file 2 — Additional file 2: Plate S2. Intact bed net is being used for unintended purpose such as bag for animal feed or hay near home (Photo courtesy: Abebe Asale). [file 12936_2021_3996_MOESM2_ESM.docx]
